# Recent Advances in Monitoring Stem Cell Status and Differentiation Using Nano-Biosensing Technologies

**DOI:** 10.3390/nano12172934

**Published:** 2022-08-25

**Authors:** Wijin Kim, Eungyeong Park, Hyuk Sang Yoo, Jongmin Park, Young Mee Jung, Ju Hyun Park

**Affiliations:** 1 Department of Biomedical Science, Kangwon National University, Chuncheon 24341, Gangwon-do, Korea; 2 Department of Chemistry, Kangwon National University, Chuncheon 24341, Gangwon-do, Korea; 3 Kangwon Radiation Convergence Research Support Center, Kangwon National University, Chuncheon 24341, Gangwon-do, Korea

**Keywords:** stem cell differentiation, single-cell level monitoring, single-cell RNA sequencing, optical spectroscopy, fluorescence, Raman, SERS

## Abstract

In regenerative medicine, cell therapies using various stem cells have received attention as an alternative to overcome the limitations of existing therapeutic methods. Clinical applications of stem cells require the identification of characteristics at the single-cell level and continuous monitoring during expansion and differentiation. In this review, we recapitulate the application of various stem cells used in regenerative medicine and the latest technological advances in monitoring the differentiation process of stem cells. Single-cell RNA sequencing capable of profiling the expression of many genes at the single-cell level provides a new opportunity to analyze stem cell heterogeneity and to specify molecular markers related to the branching of differentiation lineages. However, this method is destructive and distorted. In addition, the differentiation process of a particular cell cannot be continuously tracked. Therefore, several spectroscopic methods have been developed to overcome these limitations. In particular, the application of Raman spectroscopy to measure the intrinsic vibration spectrum of molecules has been proposed as a powerful method that enables continuous monitoring of biochemical changes in the process of the differentiation of stem cells. This review provides a comprehensive overview of current analytical methods employed for stem cell engineering and future perspectives of nano-biosensing technologies as a platform for the in situ monitoring of stem cell status and differentiation.

## 1. Introduction

Stem cells can be distinguished from somatic cells by their unique abilities to self-perpetuate and differentiate into various cell types consisting of adult tissue or organ. Stem cell behaviors, including self-renewal and differentiation, are significantly affected by a multitude of physicochemical cues, such as cell–cell and cell–extracellular matrix interactions [1], topography, and stiffness of the matrix [2,3], and cellular signaling induced by soluble cues, such as cytokines and growth factors [4,5]. These complexities in lineage commitments can result in the heterogeneity of differentiation in certain stem cell populations. Despite technical advances in controlling the developmental processes of stem cells, the differentiation into unintended lineages and the existence of undifferentiated stem cells are regarded as major obstacles to the clinical application of stem cells in regenerative medicine. In stem cell transplantation therapies, unexpected cell types can emerge during differentiation. These might cause critical problems, such as tumorigenesis [6,7].

To meet clinical needs, the exhaustive monitoring of biodistribution, engraftment, viability, and differentiation into a target lineage of transplanted stem cells is required to ensure bio-safety and to improve their therapeutic efficacy [8,9]. Up-to-date, different analysis methods of lineage-specific marker expression, such as immunostaining, Western blot, flow cytometry, and quantitative polymerase chain reaction (qPCR), have been widely used to characterize and monitor the differentiation process of stem cells [10,11]. Nevertheless, such conventional methods have some limitations due to their requirement of destructive steps, such as cell fixation and lysis, which can disrupt the spatial information in the differentiating cell population, thereby impeding longitudinal tracking from parental cells to their progenies [12,13,14]. A limitation to precise single-cell-level analysis during in vitro and in vivo differentiation due to the low resolution of the abovementioned methods is another important problem encountered in the in situ monitoring of stem cell differentiation. These limitations have compelled us to develop novel analytical methods that enable the highly sensitive in situ monitoring of stem cell differentiation at the single-cell level without any destructive steps.

The main purpose of this review is to recapitulate the latest advances in the development of technologies for tracking the lineage commitment of these stem cells, along with the necessity of monitoring their status and differentiation for clinical applications. In particular, we specifically discuss methods for the in situ monitoring of stem cell differentiation using nano-biosensing platforms, such as fluorescence-based nanotechnologies and Raman spectroscopy.

## 2. Clinical Application of Various Stem Cells and Necessity of Monitoring Differentiation Process

**Figure 1:**
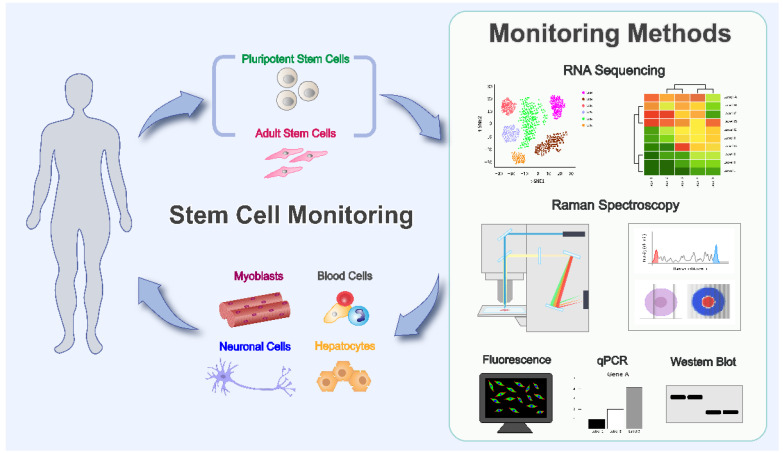
Schematic of the process of cell therapy using various stem cells and methods of tracing the lineage of these stem cells.

For clinical application, two types of stem cells have been widely studied: (1) mesenchymal stem cells, a type of adult stem cell that includes bone marrow-derived mesenchymal stem cells (BM-MSCs), dental pulp stem cells, (DPSCs), and adipose-derived stem cells (ADSCs); (2) pluripotent stem cells, such as embryonic stem cells (ESCs) and induced pluripotent stem cells (iPSCs). Here, we elaborate the use of these stem cells in regenerative medicine for disease treatments and the necessity of monitoring and controlling their differentiation processes for clinical applications (Figure 1).

### 2.1. Mesenchymal Stem Cells

In a series of historic studies in the 1960s and 1970s, Friedenstein et al. identified MSCs as a fibroblast-like non-hematopoietic population that could differentiate into bone in the bone marrow [15,16]. Since then, MSCs have also been isolated from other tissues such as umbilical cord blood [17,18], peripheral blood [19,20] skin and muscle [21], dental pulp [22,23], lung [24], and adipose tissue [25]. Many comparative studies have suggested that MSCs isolated from different tissues share common properties such as the expression of MSC-specific genes and differentiation potential toward specific lineages despite slight differences in the population numbers, growth rate, and therapeutic outcomes [26,27,28]. In addition to their capabilities of self-renewal and differentiation into multi-cell types, MSCs possess the abilities of migrating toward injured tissues (called a homing effect [29]), activating resident cells, and modulating immune responses via paracrine action [30,31]. These versatile properties enable MSCs to be utilized as an appropriate resource for regenerative medicine.

As a result of a search using “mesenchymal stem cells” as the keyword in ClinicalTrials.gov, a total of 907 clinical trials, including completed cases, were found to be registered in different phases. The indications registered for MSC-transplantation therapy include neurological disorders, such as spinal cord injury [32], multiple sclerosis, and stroke [33,34], bone and cartilage diseases, such as osteoarthritis and rheumatoid arthritis [35,36], and cardiovascular diseases [37]. Because of their differentiation potential, the therapeutic effect of MSC transplantation was attributed to the differentiation of MSCs engrafted in the injured area into damaged cells and the subsequent tissue regeneration at the early stage of clinical trials [38]. However, other studies have revealed that in vivo engraftment and differentiation into the target lineage of transplanted MSCs is usually very inefficient for having a therapeutic impact, suggesting that the main effect of MSC transplantation might not be due to their differentiation [39,40]. Preferably, many reports have demonstrated that paracrine factors secreted from transplanted MSCs play a crucial role in tissue repair and regeneration [41,42].

In addition to the ambiguity of the mode-of-action of MSC transplantation therapies, safety issues regarding the quality control of MSCs have been debated. Other failure cases have also been reported in many clinical trials despite many successful outputs of preclinical studies and small-scale clinical trials at the laboratory level [43,44]. One of the most important factors attributed to the clinical failure of these therapies is the heterogeneity in the cell population and the differentiation capability of the MSCs. Many studies have demonstrated that MSCs obtained from different donors exhibit significant discrepancies in the proliferation rate and potential for differentiation into specific lineages, resulting in deviations in the clinical efficacy of MSC therapies [45,46]. The heterogeneity of MSCs is affected by not only donors but also by the tissue source, cell isolation, culture conditions, preservation, cell populations, and the passage number of in vitro cultures [47,48,49]. The functional heterogeneity of transplanted MSCs can provoke poor engraftment and uncontrolled differentiation, resulting in not only insignificant therapeutic efficacy but also severe side effects. The potential side effects of MSC transplantation include abnormal immune responses, malignant transformation, and prothrombotic disorder [50,51].

To overcome the potential risks of MSC therapies, it is crucial to monitor the cell characteristics and differentiation. In addition to the abovementioned conventional methods such as immunostaining, Western blotting, qPCR, and flow cytometry analysis, another generally accepted method is to examine the metabolites specific to each differentiated cell type derived from MSCs, such as calcified matrices of osteoblasts [52], lipid droplets of adipocytes [53], and sulfated proteoglycans of chondrocytes [54,55]. However, these methods are destructive, with serious limitations in the analysis of in vivo differentiation. For more precise identification of the heterogeneous cell population derived from MSCs, the development of non-destructive analytical techniques capable of monitoring multiple cell type-specific markers simultaneously at the single-cell level is strongly required.

### 2.2. Embryonic Stem Cells and Induced Pluripotent Stem Cells

Pluripotent stem cells (PSCs) can proliferate unlimitedly and differentiate into all kinds of lineages consisting of adult tissue. ESCs and iPSCs are included in the category of PSCs. Since the first human ESC line was established by James Thomson and colleagues [56], the clinical application of ESCs has been explored for several incurable diseases, such as age-related macular degeneration, Parkinson’s Disease, and spinal cord injury [57]. To overcome the ethical dilemma of ESC-based therapy regarding the destruction of a human embryo and the requirement of numerous eggs in the establishment of an ESC line, a novel study delivered four specific factors to terminally differentiated somatic cells and reprogrammed them into a new type of PSCs called iPSCs [58]. Although a study suggested the clinical application of human iPSCs [59], they face many obstacles, such as the tumorigenicity and heterogeneity encountered by ESCs as well.

The risk of tumorigenicity due to the remaining undifferentiated cells is considered to be the most significant problem in the clinical application of PSC technology. Unlimited self-renewal is an important advantage in that enough cells for transplantation can be easily obtained. However, this property also results in a crucial problem because undifferentiated PSCs tend to proliferate infinitely even in vivo. Even if a few residual undifferentiated PSCs are administrated into a patient’s body, they could induce the formation of teratoma, a type of germ tumor that contains all three germ lineages simultaneously [60]. Tumorigenesis might also arise from incorrectly patterned PSCs. For example, it has been reported that the epigenetic variation among human iPS cell lines is correlated with the differentiation capacity, indicating that a PSC line with certain epigenetic characteristics is inevitably incomplete in the differentiation into a specific lineage. The resulting differentiated cells may induce tumorigenesis when transplanted in vivo [61]. Aside from tumorigenicity, the functional heterogeneity among different PSC lines due to epigenetic variations might also impede quality control for uniform and predictable therapeutic efficacy.

For these reasons, precise screening is strongly required to satisfy the safety standards for the clinical application of PSC-based therapy. To select maturely differentiated cells or eliminate incorrectly differentiated PSCs, single-cell labeling techniques using monoclonal antibodies for cell surface markers have been studied. In clinical studies for treating ocular disorders, Nishida and colleagues found a novel surface marker for the elimination of both undifferentiated human iPSCs and non-corneal epithelial cells (CECs), including other cell types of iPSC-derived retinal lineage. As a result, the risk of tumorigenesis could be reduced in iPSC-derived CEC transplantation therapy [62,63]. However, these methods still could not completely eliminate undifferentiated and/or incorrectly differentiated PSCs. Therefore, as in the case of MSCs, the development of an advanced in situ monitoring system for PSC differentiation is necessary.

## 3. Latest Technological Advances in Monitoring Stem Cell Status and Differentiation

### 3.1. Tracing Differentiation of Stem Cells at the Single-Cell Level Using Single-Cell RNA Sequencing (scRNA-seq)

With advances in molecular biology, the states of differentiating cells can be identified by investigating the expression of marker genes using immunostaining or qPCR analysis. At present, scRNA-seq capable of massive gene expression profiling at the single-cell level provides novel opportunities to analyze stem cell heterogeneity [64,65]. scRNA-seq enables the simultaneous investigation of transcripts in numerous individual cells, which allows for the tracing of the differentiation trajectory in a heterogeneous cell population. In addition, scRNA-seq can provide precise information about ‘off-target’ cell types that emerge during differentiation. Although conventional immunostaining is useful for identifying target cells generated through differentiation using lineage-specific markers, it is difficult to distinguish non-differentiated cells and off-target cells, which are cell types differentiated into other undesired lineages [66]. Reconstructing differentiation through scRNA-seq can provide opportunities to ameliorate the differentiation protocol. Some recent studies have mapped differentiation trajectories and found crucial molecular markers related to the bifurcation of lineages. By investigating a transcriptomic signature during mesendoderm to definitive endoderm (DE) development from the pluripotent state of human ESCs by scRNA-seq, Chu et al. identified a novel transcriptional regulator that governs the fate of ESCs into DE [67]. The single-cell mapping of the lineage bifurcation in the ectodermal differentiation of ESCs has revealed detailed profiles of distinct transcription factors in each lineage that emerged during early brain development, such as forebrain and mid/hindbrain lineages [68,69]. These results can provide strategies for the precise control of differentiation by stimulating target lineages while suppressing off-target lineages.

#### 3.1.1. Cell Fate Mapping by Cellular Barcoding

Recently, cellular barcoding technology has emerged as an efficient tool for the precise lineage tracing of stem cells by scRNA-seq [70]. In this strategy, the genome of each individual cell is tagged with a specific DNA sequence of a given number of base pairs. According to the length of the DNA sequence, an almost infinite number of cells can be barcoded with a distinct heritable DNA tag. The barcoding of individual cells facilitates lineage reconstruction by identifying the progeny of a particular cell labeled with a specific tag. To introduce heritable genetic barcodes, some strategies have been utilized. The most widely used method so far relies on the manipulation of the vector pool encoding unique DNA sequences, such as viruses and plasmids [71,72]. Each vector can deliver the DNA tag into an individual cell through viral or non-viral transfection. The transfected cell is thereby labeled by the unique DNA barcode [73,74,75,76]. Recent advances in CRISPR-based genome editing technologies have facilitated efficient barcode generation. The initial strategy of CRISPR/Cas9-mediated cellular barcoding relied on a repair mechanism of Cas9-induced double-strand breaks in genomic DNA by nonhomologous end-joining with a subsequent introduction of short random insertions and deletions (INDELs) at the repair loci [77]. These random mutations by INDELs at a parental genome play the role of a barcode for distinguishing cells. To increase barcode diversity, Kalhor et al. proposed a strategy of evolving DNA barcodes that can alter their genetic code gradually [78]. In this system, a guide RNA (gRNA) is engineered to target its own locus introduced in the genome during the delivery of external genes encoding the gRNA. As a result, a mutation is created within the gRNA genomic locus. Subsequently, new gRNA expressed from the mutated locus leads to another mutation at its own gRNA locus. During each cell cycle, a series of these mutations generate highly diverse and evolving DNA barcodes that can be used to be deciphered by scRNA-seq.

#### 3.1.2. Challenges of scRNA-seq-Based Differentiation Tracing

Despite its feasibility as a tool for tracing the stem cell differentiation trajectories and mapping cell fate, scRNA-seq has some limitations, including a considerable process time and cost [79,80]. One key limitation is that most scRNA-seq procedures require the disruption of tissue integrity and cell destruction, which can result in the loss of the spatial information in heterogenous cell populations [81,82]. During stem cell differentiation, each cell communicates with neighboring cells. Physicochemical interactions that trigger lineage commitment are significantly affected by an organized microenvironment. Spatial information that indicates which types of neighboring cells the cell to be tracked is in contact with (e.g., cells differentiated into the target lineage or off-target cells) is also crucial for the lineage commitment of stem cells, as is genomic or transcriptomic information. Thus, the loss of spatial information due to the destructive nature of scRNA-seq makes it difficult to trace cell fate and find crucial factors affecting differentiation into a specific lineage.

The requirement of genetic manipulation in DNA-barcoding before scRNA-seq-based lineage tracing and its low efficiency are other important technical obstacles. To transduce hard-to-transfect cells, such as human PSCs, with a barcode-expressing construct, a high multiplicity of infection and multiple rounds of infection with retrovirus or lentivirus are required, which can result in severe cell death [83]. In the case of CRISPR barcoding methods, the potential off-target genetic mutations due to residual endonuclease activity might be an important reason for disturbing the developmental dynamics of stem cells.

In addition, the incomplete detection of unique barcode sequences, which can arise from a low signal-to-noise ratio of barcode readout or the endogenous knockout of transgenes, can distort the results of differentiation tracing [73]. Failure to capture and label even only a small fraction of the entire cell population might also skew the interpretations of scRNA-seq data [84]. Genetically unrelated cells are labeled with identical barcode sequences (barcode homoplasy), which can also cause the failure of cell fate monitoring in heterogeneous stem cell differentiation.

### 3.2. Fluorescence Spectroscopy to Monitor Stem Cell Differentiation

In the field of bioscience, the fluorescence imaging technique is widely used as a valuable tool to monitor the expression of target proteins, cellular processes, and cell dynamics [85,86]. It can also be used to visualize single cells, tissue, organs, or a whole body in real-time [87,88]. Fluorophore or fluorescent proteins are essential for applying this method to the detection of a target protein. In this method, when excitation photons are irradiated, emission photons are emitted from the fluorophore [89,90]. Fluorescence studies using immunohistochemistry and nanomaterials have been performed.

#### 3.2.1. Immunocytochemistry (ICC)

ICC is the most used fluorescence protocol. It can evaluate the populations of stem cells. In this method, the secondary antibody with fluorescent tags detects the primary antibody bound to the target protein. For successful immunocytochemistry, it is important to select antibodies that can specifically bind to the target [91]. Based on previous studies, various proteins, such as TRA-1-60 [92], SSEA-4 [93,94], Sox2 [95], Oct4 [96,97], and Sushi-containing domain 2 [98], are markers for PSCs. Antibodies suitable for such proteins are usually used for fluorescence imaging [99]. Table 1 summarizes the studies that have monitored the differentiation process of stem cells over the past three years using immunocytochemistry [95,98,100,101,102,103,104,105,106,107,108,109,110,111,112,113,114,115,116,117,118,119,120,121,122,123,124,125,126,127,128,129,130,131,132,133,134,135,136]. ICC is a non-destructive method, different from Western blot.

#### 3.2.2. Nanomaterials in Fluorescence-Based Biosensing

Nanomaterials have optical properties, such as photostability and the control of excitation and emission wavelengths [137]. Fluorescent nanoparticles can be monitored for a long time due to their long stability in cells. Graphene quantum dots (GQDs), a type of nanomaterial, have been used as fluorescent materials because they can maintain excellent photoluminescent and photostability [138,139]. To improve their biocompatibility, GQDs have been combined with biochemically inert polyethylene glycol (PEG) [140]. Ji et al. [140] reported that 320 μg/mL of PEG-GQDs did not affect the differentiation from rat neural stem cells to neurons or glial cells and that PEG-GQDs composites showed adequate bioimaging capabilities when they were internalized into neural stem/progenitor cells. The synthesis of fluorescent polymers with high stability and a high quantum yield has also been reported [141,142,143]. Jang et al. [143] reported that aggregation-induced emission nanoparticles (AIE-NPs) can penetrate greatly into cells. AIE-NPs in cells can be retained for a long time without altering the neuronal proliferation, differentiation, or viability in vitro. AIE-NPs labeled neuronal grafts were tracked for one month in mouse brain striatum at various time points after transplantation. Choi et al. [144] found that living human MSCs (hMSCs) could be differentiated into osteogenic lineage using polydopamine-coated gold (Au) nanoparticles (Au@PDA). To recognize a target mRNA, a hairpin DNA (hpDNA) strand with a fluorescent tag was immobilized at the PDA shell. Fluorescent signals were quenched by Au@PDA and recovered when the hpDNA was dissociated from the Au@PDA by the target miRNA. Au@PDA–hpDNA displayed fluorescence in hMSCs differentiated into primary osteoblasts. Using this phenomenon, the differentiation process could be monitored. In addition, some recent studies using DNA nanotechnology in combination with fluorescence spectroscopy as a tool for biosensing have been reported [145,146,147].

Although fluorescence signaling using monofunctional nanoparticles could be efficient for stem cell monitoring, some limitations still remain. Accordingly, studies on the fabrication and application of multifunctional nanoparticles have been widely conducted. Nanomaterial-based fluorescence dyes can be used in drug delivery and transplant treatment through monitoring while tracking in vivo mechanisms [148,149,150,151]. Li et al. [151] reported on a novel near-infrared (NIR) light-activated nanoplatform for the remote control of cell differentiation and the real-time monitoring of differentiation simultaneously. This was attained by encapsulating a photoactivatable caged compound (DMNPE/siRNA) and combining a matrix metalloproteinase 13 (MMP13) cleaved imaging peptide-tetraphenylethylene (TPE) unit conjugated with mesoporous silica-coated up-conversion nanoparticles (UCNPs). When irradiated with NIR light, the photoactivated caged compound was activated and the siRNA was released from the UCNPs, enabling the controlled differentiation of stem cells by light. The MMP13 triggered by osteogenic differentiation effectively cleaved the TPE probe peptide, enabling the real-time monitoring of stem cell differentiation by aggregation-induced emission.

Gold nanoparticles (AuNPs) have been widely studied in medical fields, such as imaging, drug delivery, and theragnostic systems [152]. Wu et al. [153] developed multifunctional AuNPs to control cell fate and simultaneously detect the osteogenic differentiation of hMSCs in real time. AuNP-polyethyleneimine-peptide-fluorescein isothiocyanate/small molecule-interfering RNA (AuNP-PEI-peptide-FITC/siRNA) nanocomplexes to control the osteogenic differentiation of hMSCs could be silenced by the peroxisome proliferator-activated receptor γ (PPARγ), an adipogenesis-related gene. By measuring the activity of the MMP13 enzyme produced during osteogenic differentiation through the recovery of FITC fluorescence, it was demonstrated that AuNP nanocomplexes could control cell differentiation. They could be used as a nanoprobe for the real-time detection of the osteogenic differentiation of hMSCs.

### 3.3. Profiling and Tracing of Stem Cell Differentiation Using Raman Spectroscopy

Raman spectroscopy is a powerful method that can characterize cell information at the molecular level while obtaining images of stem cells at the same time [154,155,156]. It is based on inelastically scattered photons with different frequencies from excitation photons. It is the change in the wavelength of the scattered photon that provides the chemical and structural information [157]. Therefore, Raman spectroscopy reflects the fingerprint region of the target [158,159,160,161]. Raman spectroscopy is suitable for the long-term monitoring of cellular processes due to its advantages such as light stability [161] and no need for antibodies [162].

#### 3.3.1. Raman Spectroscopy for Identification of Stem Cell Differentiation

**Figure 2:**
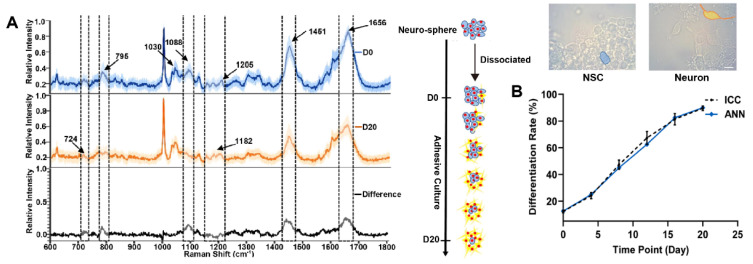
(**A**) Raman spectra of stem cells on day 0 (blue) and day 20 (yellow). The black spectrum shows differences. (**B**) Evaluation of differentiation rate using machine learning based on Raman spectra. Adapted with permission from Ref. [163]. Copyright 2021 American Chemical Society.

Monitoring the stem cell differentiation process by Raman spectroscopy has been very actively studied in the last decade. Generally, the Raman spectrum of a cell is described as a typical fingerprint including the nucleic acid (at 720–820 and 1080–1100 cm^−1^), lipid (1310 and 1440 cm^−1^), protein (1030, 1170–1210, and 1610–1660 cm^−1^), and amino acid residue (e.g., phenylalanine, 1002 cm^−1^) (Figure 2) [163,164,165]. However, the position and intensity of the Raman bands are different for each cell because the cell components are different. The analysis of a Raman spectrum of a stem cell that contains various chemical compounds is very complicated. Therefore, multivariate analyses, such as principal component analysis (PCA), have been applied to identify and distinguish the differentiation of stem cell processes [166,167,168].

Lazarevic et al. [166] investigated stem cell differentiation to adipogenic, chondrogenic, and osteogenic lineage using micro-Raman spectroscopy. The relative intensities of bands corresponding to nucleic acids and proteins can distinguish the state of adipogenic differentiation. During adipogenic differentiation, the intensities of the nucleic acid bands decrease, while those of the protein and lipid bands increase. The amounts of protein and proteoglycan increased during the chondrogenic differentiation. In the case of osteogenic differentiation, the spectral difference between human periodontal ligament stem cells (hPDLSCs) and osteoblasts was due to decreases in the band intensities of amino acids and lipids and increases in the band intensities of carbonates and phosphates.

During the differentiation of stem cells into neurons, the intensity of the lipid band decreases, while that of the protein increases. Based on these results, it was found that 12% of stem cells on day 0 already started toward neuron differentiation, whereas 10% of the cells did not differentiate after 20 days. To improve the prediction of the differentiation rate, an artificial neural network (ANN) was used. An ANN is a machine learning method that can provide a non-invasive classification of cell types at the single-cell level [163]. Compared to the Raman spectrum of a neuron cell, a band at 480 cm^−1^ assigned to glycogen appeared in the Raman spectrum of the iPSC [167].

Undifferentiated myoblast cells and myoblast cells differentiated at three different stages can be easily distinguished using deep UV resonance Raman spectroscopy combined with chemometric techniques [168]. Mandair et al. [169] reported that bone tissue formed in an osteogenic cell culture exhibited progressive matrix maturation and mineralization. However, they could not fully replicate the high degree of collagen fibril order found in native bones.

Alraies et al. [170] reported on the discrimination between different DPSCs populations by DNA and protein using the averaged Raman spectrum of DPSCs. Simonović et al. [171] investigated the differentiation of three dental stem cells (apical papilla (SCAP), dental follicle (DFSC), and pulp (DPSC)) using the relative intensity ratio of the tryptophan band versus nucleic acid observed in their Raman spectra.

#### 3.3.2. Raman Spectroscopy for Imaging Stem Cell Differentiation

Raman spectroscopy can be used to add image contrast to visualizing structures and dynamics in living systems and materials. Chemical composition is most commonly used to provide contrast for determining the spatial distributions of chemical components [172].

Ravera et al. [173] reported that Raman micro-spectroscopy can contribute to the understanding of biochemical evolution underpinning the cellular progression from an undifferentiated state to a condensation stage and then to a terminally differentiated state. To monitor the process of MSC differentiation into chondrocytes in vitro, Raman micro-spectroscopy was used, providing a holistic molecular picture of cellular events governing the differentiation. They found that the characteristic signatures of several specific macromolecules of the extracellular matrix (ECM) were identifiable in the early stages of condensation, including collagen type II, proteoglycans, adhesion molecules, and several other proteins, whereas, in the latter stages, the elaboration of the lipidic content of the ECM appeared to be the most significant. Using Raman imaging, De Bleye et al. [174] monitored the synthesis of ECM and its progressive mineralization during the osteogenic differentiation process.

**Figure 3:**
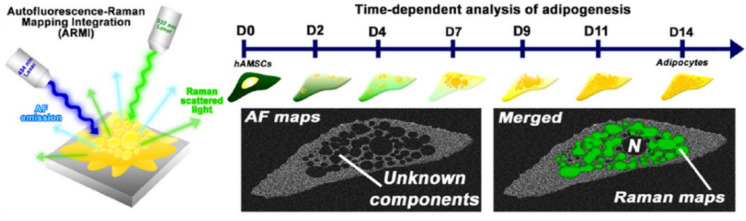
Schematic illustration of AF-Raman mapping analysis. Reprinted with permission from Ref. [175]. Copyright 2021 Elsevier.

Raman spectroscopy can detect the osteogenic differentiation process of stem cells more sensitively than other chemical-based staining assays. Suhito et al. [52] reported on a new method capable of the in situ label-free quantification of stem cell differentiation into multiple lineages, even at a single-cell level. Based on Raman images, they found that the osteogenesis of hADSCs could be determined and quantified after 9 days of differentiation. This result is a week earlier than the typical Alizarin Red S (ARS) staining method. They [175] also reported a novel autofluorescence-Raman mapping integration analysis for the ultra-fast label-free monitoring of adipogenic differentiation (Figure 3). In this method, autofluorescence (AF) imaging based on endogenous fluorophores in cells enables the rapid visualization of the cell morphology and cytosolic microstructure, while Raman mapping based on a specific molecular signature can precisely identify the biological molecules of interest. This method can avoid the erroneous characterization of individual cells in the same batch or different batches.

**Figure 4:**
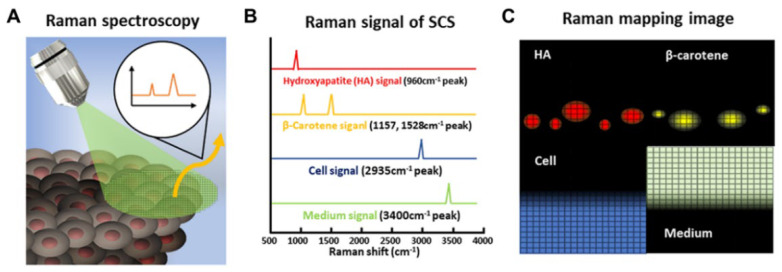
(**A**) Schematic illustration of 3D Raman mapping-based analytical method. (**B**) Characteristic peak of HA, β-carotene, cell, and medium. (**C**) 3D Raman mapping using specific peaks. Adapted with permission from Ref. [176]. Copyright 2021 American Chemical Society.

Kim et al. [176] reported a 3D Raman mapping-based analytical method to identify crucial factors responsible for inducing variability in differentiated stem cell spheroids. They analyzed and monitored human DPSC spheroids based on three different Raman bands of hydroxyapatite (odontogenic differentiation marker), β-carotene (precursor of hydroxyapatite), and proteins/cellular components (cell reference) (Figure 4). Dou et al. [177] reported the diagnosis of the early-stage differentiation of mouse ESCs using Raman imaging. Raman imaging was used to characterize the spectral features of undifferentiated inner cells and peripheral cells of differentiated embryoid bodies. PCA was employed to obtain more differences and discriminate between the two cell types. Kukolj et al. [178] investigated inter-individual differences between BM-MSCs at a single-cell level by Raman spectroscopy. Despite having a similar biochemical background, fine differences in the Raman spectra of the BM-MSCs of each donor could be detected.

#### 3.3.3. Surface-Enhanced Raman Spectroscopy (SERS)

Although Raman spectroscopy can be used for the identification and imaging of stem cells, it also has some limitations due to its low cross-section [179]. The low cross-section attenuates the signal-to-noise ratio, leading to an increase in measurement time. An extended measurement time not only causes serious damage to the cells but also makes it difficult to measure a large number of cells [180]. Another limitation of Raman scattering is the difficulty of measuring subtle changes in the proteins and minerals. SERS is a promising technique that can compensate for this shortcoming. SERS is a phenomenon that can significantly enhance the Raman signal when molecules are absorbed or approached near the surface of metal nanoparticles [181]. It can detect even single molecules and is suitable for long-term monitoring [182]. Although roughed gold, silver, and copper metal surfaces have been used as typical SERS active substrates [183], recently, a SERS substrate was expanded with semiconductors, graphene, and quantum dot, which also show strong SERS enhancement [184]. For various purposes, SERS substrates can be fabricated in different sizes, shapes, and coatings [185].

**Figure 5:**
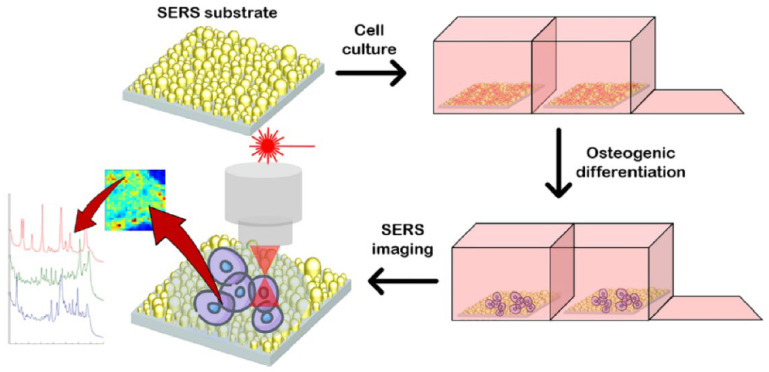
Schematic illustration of label-free SERS monitoring differentiation of BM-MSCs using gold substrate. Adapted with permission from Ref. [192]. Copyright 2021 American Chemical Society.

There are two SERS strategies—labeled and label-free methods for detection with and without biomarkers, respectively. The label-free SERS method provides rich stem cell information for identification without labeling the analytes [186,187,188,189,190,191,192]. In this method, the information of the biomacromolecules, such as the protein structure, nutrient amounts, and biological processes occurring at the cellular level that change during differentiation, can be detected. Milewska et al. [189,192] reported on the differentiation of BM-MSCs using label-free SERS. They examined the bands of cholesterol, proteins, collagen backbone, proline, calcium hydroxyapatite, and a phospholipid alkyl chain for SERS mapping (Figure 5) [192]. BM-MSCs cultured on a biocompatible nanostructured gold substrate for 7 and 21 days revealed different stages of the differentiation spectrum of individual cells, such as molecular species and chemical events on the cellular membrane [189,192].

SERS can also be applied to biological processes occurring inside stem cells [191]. The profiling of molecular changes in the nucleus of a single DPSC was monitored by AuNPs functionalized with a cell-penetrating peptide. Wang et al. [191] identified the differentiation process of DPSCs stimulated by drugs. Using label-free SERS, they found that two pivotal differentiation biomarkers, alkaline phosphatase (ALP) and dentin sialophosphoprotein (DSPP), were overexpressed during the process. The corresponding transformation of the DNA/RNA backbone vibrational modes was also observed during the differentiation process, indicating the occurrence of the replication or transcription of the DNA.

**Figure 6:**
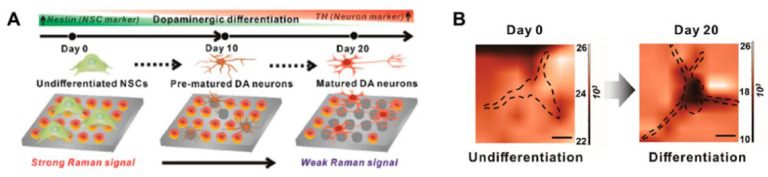
(**A**) Schematic illustration of characterization of neural differentiation using GO-hybrid nano-SERS array. (**B**) SERS mapping imaging before and after neural differentiation. Adapted with permission from Ref. [194]. Copyright 2020 American Chemical Society.

Indirect SERS methods have also been widely studied because label-free SERS spectra are too complex for analyzing specific targets [179,193]. Choi et al. [194] developed a graphene oxide (GO)-hybrid nano-SERS array to detect dopamine (DA) that can characterize the differentiation of neural stem cells (Figure 6). During neural differentiation, the DA was complexed with a DA-binding DNA aptamer conjugated with Raman dye and released from the hybrid nano-SERS array. As a result, the degree of differentiation of the neural stem cells was evaluated from the decreased SERS signal. This developed SERS-based detection method can investigate single-cell signaling pathways associated with DA or other neurotransmitters and their roles in neurological processes.

Gold nanostructures (AuNPs), such as nanocages and nanostars, can be easily adjusted to expand the localized surface plasmon resonance (LSPR) to near-infrared (NIR). Nanocages facilitate efficient drug delivery to stem cells through a biocompatible interior hollow space [195]. Cao et al. [196] reported an ultrasensitive SERS method for the long-term detection and imaging of miR-144-3p in the osteogenic differentiation of BM-MSCs. They detected miR-144-3p, an osteogenic differentiation biomarker of BM-MSCs, using Au nanocage-hairpin DNA. They found that these nanoprobes were capable of the long-term tracking of the dynamic expression of miR-144-3p (21 days) in differentiating BM-MSCs.

In addition, nanostars not only have many sharp tips that can produce highly sensitive signals by forming strong LSPR, but also have a large surface area, allowing various biomolecules to connect to the surface [197,198]. Hua et al. [197] fabricated a gold nanostar (Au-Star)-based second near-infrared window (NIR-II) fluorescence/SERS dual-modal imaging probe for the labeling and precise tracking of stem cells. Using this imaging approach, stem cells in hypodermic and myocardial infarction models can be tracked with high resolution and depth-independent imaging capabilities.

### 3.4. Other Methods for Monitoring of Stem Cell Differentiation

Although optical spectroscopy is an attractive tool for monitoring stem cell differentiation, it may cause photodamage of cells [180]. To avoid the photodamage of cells, it is necessary to extend the range of the light source or optimize the procedure to reduce the measurement time. Here, we introduce other spectroscopic methods for monitoring stem cell differentiation.

#### 3.4.1. Infrared (IR) Spectroscopy

IR spectroscopy is a powerful tool to characterize each cell type and state based on specific molecular properties represented by sensitive IR spectroscopic fingerprints. [199]. Since the energy of the excitation light source of IR spectroscopy is lower than that of Raman or fluorescence spectroscopy, it has a lower risk of photodamage [200]. IR spectroscopy can distinguish between stem cells and their derivatives.

Wang et al. [201] developed a single-cell Fourier transform infrared (FTIR) micro-spectroscopy based on the method for the quantitative evaluation of cellular heterogeneity by calculating the cell-to-cell similarity distance of IR spectral data. They obtained spectral mapping images of the hMSC differentiation based on 2940–2910 cm^−1^ (fatty acid), 1670–1600 cm^−1^ (protein), and 1133–1033 cm^−1^ (nucleic acid) and revealed that IR phenotypes might reflect dynamic heterogeneity changes in the cell population during adipogenic differentiation. This is enough to measure the MSC, which has an average diameter of 18 μm. Gieroba et al. [202] used macro attenuated total reflection (ATR)-FTIR spectroscopic imaging for the analysis of a ceramic-based biomaterial (chitosan/β-1,3-glucan/hydroxyapatite). According to their study, this spectroscopic approach is very suitable for studying the formation of new bone tissue and ECM components because sample staining and demineralization are not required. Thus, this approach is rapid and cost-effective.

#### 3.4.2. Second Harmonic Generation (SHG) Scanning

SHG is a nonlinear optical process that is sensitive to the symmetry of media [203]. SHG microscopy has been applied to various specimens, including biological samples such as collagen, myosin, and microtubules. SHG has the advantages of no bleaching, no blinking, no signal saturation, and a high signal-to-noise ratio compared to fluorescence. Qi et al. [204] demonstrated the possibility of the stem cell internalization of boron-doped graphene quantum dots (B-GQDs) as an SHG probe and showed no hindering of the central physiological activities of the stem cells, such as differentiation.

Ibrahim et al. [205] performed structural analysis using extracellular collagen alignment and the mineral density in bone tissue engineered samples to evaluate the osteogenic maturation of human hASCs. They demonstrated that altering the physical environment and introducing a blood supply can enhance the maturity of the bone to which these cells form. Kourgiantaki et al. [206] demonstrated that grafts based on porous collagen-based scaffolds, similar to biomaterials utilized clinically in induced regeneration, can deliver and protect embryonic neural stem cells (eNSCs) at spinal cord injury (SCI) sites, leading to the significant improvement of locomotion recovery in an experimental mouse SCI model.

#### 3.4.3. Hyperspectral Spectroscopy

Hyperspectral imaging (HSI) integrates conventional imaging and spectroscopy and is a label-free detection method [207]. Ogi et al. [208] reported on a label-free observation method for stem cell research that can classify neurons and glia in neural stem cell cultures using HSI microscopy combined with machine learning. Metha et al. [209] reported on a novel methodology using HSI combined with spectral angle mapping-based machine learning analysis to distinguish differentiating human ADSCs from control stem cells.

## 4. Conclusions

Uncontrolled differentiation is one of the major obstacles in stem cell transplantation therapies. For preventing phenotypical alterations, such as tumorigenesis and physiological heterogeneity, methods capable of accurate in vivo characterization of transplanted stem cells at the single-cell level are required. Recently, various strategies based on single-cell transcriptomics, such as scRNA-seq, have been developed to monitor the status of stem cells and predict their differentiation trajectories. Despite its excellence in facilitating cell fate mapping by providing enormous information about the transcriptome of a large population at the single-cell level, scRNA-seq has a crucial limitation in that it contains a cell disruption step, hindering the spatial information of particular cells as well as the in situ tracking of differentiation. As non-destructive alternatives, optical spectroscopies such as fluorescence, Raman, IR, SHG, and HSI, have been studied for monitoring with high sensitivity and accuracy at the single-cell level. Raman is a label-free method that can screen differentiation and obtain chemical information at the molecular level of stem cells, which can be used to discriminate stem cells according to their differentiation state. SERS has an enhanced Raman signal, thereby facilitating the detection of biomolecules, such as neurotransmitters, with more sensitivity. By examining Raman and SERS spectra with PCA and machine learning, many attempts have been made to determine the degree of stem cell differentiation and cluster each differentiation state. Although other limitations of spectroscopy-based techniques, such as long measurement times and low sensitivity in some cases, still remain, recent studies have attempted to overcome these limitations by combining two or three spectroscopy methods simultaneously or utilizing artificial intelligence technology. In the future, technologies discussed in this review can be used as an advanced monitoring system for in vivo and in vitro studies for clinical applications using stem cells.

## Figures and Tables

**Table 1:** Recent studies related to stem cell differentiation using immunocytochemistry.

Stem Cell	Cell Source	Target Lineage
Adipose-derived stem cell (ADSC)	Human ADSC	Human Schlemm’s canal cell [100]
Dental pulp stem cell (DPSC)	Human DPSC	Motor neuron cell [101]
	Human DPSC	Osteogenic cell [102,103]
	DPSC, dental follicle stem cells, periodontal ligament stem cell	Osteogenesis [104]
Embryonic stem cell (ESC)	Mouse ESC	Neural crest cell [105]
	Mouse ESC	Neuron [106]
	Mouse ESC	Embryoid body [107]
	Human ESC	Retinal pigment epithelial [108]
	Human ESC	Somatic cell [98]
Induced pluripotent stem cell (iPSC)	Human iPSC	Neural crest stem cell [95]
	Human peripheral blood mononuclear cell	iPSC [109]
	Human iPSC	Neuron [110,111]
	Human iPSC	Neuron [112]
	Human iPSC	β-cell [113]
	Human iPSC	Cardiomyocyte [114]
Mesenchymal stem cell (MSC)	Human adipose-derived (AD)-MSC	Cardiomyocyte [115]
	Human umbilical cord (UC)-MSC	Retinal pigment epithelial [116]
	Human UC-MSC	Chondroprogenitor [117]
	Human bone marrow (BM)-MSC	Neuron [118]
	Mouse MSC	Bone [119]
	Rat BM-MSC	Neurosphere [120]
	Rat BM-MSC	Bone [121]
	Human MSC	Nucleus pulposus-like cell [122]
	Mouse BM-MSC	β-cell into pancreatic lineage [123]
	Human AD-MSC	Pancreatic cell [124]
	Human MSC	Osteogenic and chondrogenic lineage [125]
	Human MSC	Cardiac cell [126]
	Rat BM-MSC	Adipogenic and chondrogenic cell [127]
Neural stem cell (NSC)	Rat NSC	oligodendrocyte [128]
	NSC	Neuron [112,129,130,131]
	Premigratory neural crest stem cell	Enteric neuron [99]
	Monkey NSC	Neuronal cell, glial cell [132]
	Rat NSC	Neuron [133]
	Human NSC	Neuron [134]
	NSC/progenitor cell	Neuron [135]
Parthenogenetic stem cell	Mouse parthenogenetic stem cell	Cardiomyocyte [136]

## Data Availability

The original contributions presented in the study are included in the article.
